# Effect of Mechanical Horse Practice as New Postural Training in Patients With Neurological Disorders: A Pilot Study

**DOI:** 10.3389/fpsyg.2019.01035

**Published:** 2019-05-08

**Authors:** Héloïse Baillet, David Leroy, Eric Vérin, Claire Delpouve, Nicolas Benguigui, John Komar, Régis Thouvarecq

**Affiliations:** ^1^Normandie Univ, UNIROUEN, CETAPS, Rouen, France; ^2^Normandie Univ, UNIROUEN, GRHV EA 3830, Rouen, France; ^3^Rouen University Hospital, Rouen, France; ^4^CRMPR Les Herbiers Rehabilitation Center, Bois-Guillaume, France; ^5^Normandie Univ, UNICAEN, CESAMS, Caen, France; ^6^National Institute of Education, Nanyang Technological University, Singapore, Singapore

**Keywords:** rehabilitation, posture, horse simulator, brain-injured patients, motor control, dynamic approach, constraints

## Abstract

**Objective:** From a dynamic system approach, this study evaluated the impact of a new training protocol *using a mechanical horse* on the postural coordination of brain-damaged patients.

**Methods:** Eighteen volunteer brain-damaged patients (i.e., post-stroke or traumatic brain injury) were recruited and randomly divided into an experimental group (horse group; *n* = 10, conventional therapy associated with horse-riding exercise on the mechanical horse for 30 min, twice a week, for 12 weeks) and a control group (*n* = 8; conventional therapy without intervention on the mechanical horse). Postural coordination was evaluated during pre- and post-tests through discrete relative phase (DRP) computation: ϕ_Head−Horse_, ϕ_Trunk−Horse._

**Results:** A significant effect of used training has been showed, *F*_(1, 15)_ = 16.6 (*p* < 0.05) for all patients, concerning the trunk/horse coordination.

**Conclusion:** This pilot study results showed the impact of this new training method on the postural coordination of these patients. After 24 sessions, the coordination of the horse group patients differed from that of the control group, showing their ability to adapt to constraints and develop specific modes of postural coordination (trunk/horse *antiphase*) to optimize their posture.

## Introduction

The dynamic approach of motor control in line with Bernstein (1967) is the theoretical framework used to interpret the findings. Bernstein's work is based on the concept that postural coordination is a necessary background component for any voluntary motor action (Newell, 1985). From this perspective, the redundant degrees of freedom of the postural system provide an adaptive means to maintain balance under the interaction effects of a variety of constraints: task, environment, and organism (Newell, 1986; Riccio and Stoffregen, 1988). Hence, pathology can be considered an intrinsic constraint of the organism that modifies the system dynamics, particularly postural coordination (Holt et al., 2010).

Most often, postural coordination analysis is performed on healthy individuals in a standing position (Bardy et al., 1999, 2002; Oullier et al., 2002; Bardy, 2004; Faugloire et al., 2005). Nevertheless, erect posture is often difficult to achieve for disabled individuals, particularly brain-damaged patients, who may thus be constrained to the sitting position. These patients (post-stroke, particularly) frequently present balance deficits, striking asymmetry of motor control, or alterations in spatial cognition, which increase the postural oscillations and decrease stability compared with healthy people (Shumway-Cook et al., 1988; Geurts et al., 2005; Varoqui et al., 2010). However, sitting posture has been less studied, despite its being a common and familiar position, notably for infants. In fact, most of the studies have focused on the development of postural control, which is the dynamic process whereby the infant learns to control the body's degrees of freedom to achieve the sitting posture (Hirschfeld and Forssberg, 1994; Hadders-Algra et al., 1996; Thelen and Smith, 1996; Harbourne and Stergiou, 2003; Heide et al., 2003). Although few studies have characterized the sitting posture of disabled patients, two important aspects have nevertheless been underlined: stability and dynamic stability, which reduces the body's motion or sway (Lanzetta et al., 2004). Trunk stability is based on correct perception of the body and the development of adequate muscle responses, which are constantly modified by the interaction of the constraints applied to the system. In non-standing positions, postural muscles are active in a craniocaudal order, with the neck muscles recruited before the trunk muscles (Hadders-Algra et al., 1996, 1998; van der Heide and Hadders-Algra, 2005). Moreover, head movements have an important role as they help to explore the environment through the visual and vestibular systems (Lanzetta et al., 2004). According to the same authors, the pelvis can be compared to a rigid body moving around a mediolateral axis and is considered as a stable support surface for the trunk. These studies have shown the importance of head, trunk, and pelvis movements in the sitting posture. Therefore, contrary to the findings of Bardy (2004) and Bardy et al. (1999, 2002), the sitting posture cannot be characterized by ankle-hip coordination but instead by the coordination between head, trunk, and hip (Forssberg and Hirschfeld, 1994; Van der Fits et al., 1998). In any case, the sitting posture, like the standing posture, is used daily and is part of the postural repertoire. This posture is a platform for other motor activities [e.g., catching an object (Van Der Fits and Hadders-Algra, 1998; Heide et al., 2003; Lanzetta et al., 2004)] (Forssberg and Hirschfeld, 1994) and for certain rehabilitation exercises, particularly in hippotherapy (Lechner et al., 2007; Beinotti et al., 2010; Giagazoglou et al., 2012; Menezes et al., 2013).

Postural coordination analysis is based on the study of an informational unit: the order parameter (Haken, 1983), which is a collective variable characterizing the coordination of many elements. This variable is mainly represented by the computation of relative phase (ϕ_*rel*_) (Kelso, 1995). In the standing position, the ϕ_*rel*_ between the angular movements of two non-homolog joints, the hip and ankle, appears to be a natural candidate for describing postural coordination (Bardy et al., 1999). The above-cited studies (e.g., see the protocol described by Bardy et al., 1999) have shown two spontaneous coordination modes—strong attractors—between hip and ankle movements during a task of target tracking: an *in-phase* pattern (ϕ_*rel*_ = 0° ± 20°; characterized by simultaneous flexion or extension of ankles and hips) for low frequencies and small amplitudes, and an *antiphase* pattern (ϕ_*rel*_ = 180° ± 20°; characterized by the flexion of one joint when the other joint is in extension) for high frequencies and large amplitudes (Bardy et al., 1999, 2002; Bardy, 2004; Oullier et al., 2006). However, the adoption of these two patterns and their modification or destabilization is assumed to be relative to learning new postural patterns (Faugloire et al., 2005) or modifications under environmental constraints (Bardy et al., 1999; Marin et al., 1999; Oullier et al., 2002, 2006).

In addition to its emotional and psychological dimensions, hippotherapy uses rhythmic equine movements for postural rehabilitation, which means that it is adapted to treating patients with neurological impairments (Lechner et al., 2003). Several studies have indicated the positive effects of this technique on muscle tone, posture, balance, and pain, as well as its psychosomatic influence on patients (Lechner et al., 2003; Meregillano, 2004; Debuse et al., 2005). Moreover, the brain's plasticity enables it to adapt to environmental pressure, experiences, and challenges, including brain damage (Johansson, 2000). Indeed, plasticity is defined as “the adjustment of the nervous system to changes in the external milieu (i.e., sensory inputs) or internal milieu (i.e., the effects of damage of the system) and appears to be mainly a property of the cerebral cortex rather than subcortical structures” (Thomas, 2003, p. 96). Consequently, a cortical reorganization of the brain is possible after injury through adapted rehabilitation (Johansson, 2011). Plasticity is limited, however, particularly in relation to the patient's age [a child's brain has greater plasticity that an adult brain (Thomas, 2003)]. Some authors have shown the sensorimotor benefits of a hippotherapy protocol after severe traumatic brain injury, specifying that “Hippotherapy represents an exciting new support for rehabilitation of neurological disorders” (Galeole et al., 2014, p. 3).

However, like any animal, the horse—which is used for hippotherapy—may exhibit unpredictable behaviors. Hence, a new tool, the mechanical horse, was created in the 1990s (Baillet et al., 2017b) and has been used in rehabilitation centers to improve the motor abilities, muscle tone, postural coordination, and energy expenditure of disabled patients. This tool can also be used as a first step toward real hippotherapy. Although very few studies can be found in the literature, certain have indicated that the mechanical horse elicits sensorimotor effects similar to those of hippotherapy (Temcharoensuk et al., 2015). In addition, a recent study characterized the energy expenditure and postural coordination of healthy subjects (riders and non-riders) during an exercise protocol on the mechanical horse (Baillet et al., 2017b). The results showed similarities between riders and non-riders for energy expenditure, but the main result indicated that expert participants had a more effective posture on the horse, contrary to non-riders.

Given the importance of protocols for the functional rehabilitation of postural coordination in disabled patients and the documentation of the beneficial effects of hippotherapy for these patients, the aim of this pilot study was to evaluate the impact of a new training protocol with the mechanical horse on the postural coordination of brain-damaged patients.

We hypothesized that 24 training sessions of mechanical horse movements would improve patient posture, coordination and stabilization on the horse and help them to resist postural disorganization when the oscillation frequencies increased.

## Materials and Methods

### Standard Protocol Approvals, Registrations, and Patient Consents

After an explanation of the study purpose, all participants gave written informed consent to participate in the study in accordance with the Declaration of Helsinki. This study was approved by the human research ethics committee of Lille University (n° 2016-1-S39). Enrollment was between March 2016 and March 2018 and the last patient completed intervention at the end of March 2018.

### Participants, Eligibility, and Randomization

Eighteen volunteer brain-damaged patients (i.e., post-stroke or traumatic brain injury) were recruited and screened for eligibility from a medical rehabilitation hospital (CRMPR Les Herbiers, France). These patients were randomly divided (a sealed envelope was open during the inclusion of each patient) into an experimental (horse group) and a control group (Table 1; Supplementary Material), according to a blinding procedure of open label study (all parties, patients, and clinicians, were aware of the training method the participant receive). The inclusion criteria were as follows: (1) diagnosis of mild to moderate brain damage (Glasgow Coma Scale >9) that was non-progressive and had persisted for at least 3 years, (2) age more than 18 years and <65 years, and (3) ability to maintain a sitting position with or without technical assistance.

**Table 1:** Clinical information of control and horse groups.

	**Control group**	**Horse group**
*N*	8	10
Gender	5 men/3 women	8 men/2 women
Age	39.9 ± 18.6	51.1 ± 13.0
Weight (kg)/Height (cm)	78.7 ± 19.2/175.3 ± 7.8	83.25 ± 17.3/177.3 ± 9.9
Pathological composition	7 post-strokes *(2 ischemic and 5 hemorrhagic)* 1 traumatic brain injury	9 post-strokes *(3 ischemic and 6 hemorrhagic)* 1 traumatic brain injury
Clinical information	3 hemiplegics *(1 left, 2 right)* 1 hemiparetic *left* 4 with a motor/balance deficit	3 hemiplegics *(2 left, 1 right)* 2 hemiparetic *(1 left, 1 right)* 5 with a motor/balance deficit

### Design

The control group (*n* = 8) was composed of 7 post-strokes and 1 traumatic brain injury patients and received only conventional therapy, without intervention on the mechanical horse. In contrast, the horse group (*n* = 10) was composed of 9 post-strokes and 1 traumatic brain injury. and received conventional therapy associated with horse-riding exercise using the mechanical horse (Baillet et al., 2017b) for 30 min twice a week, for 12 weeks (with two training sessions replaced by two mechanical horse sessions). The conventional therapy in the rehabilitation center consisted to general transfer abilities (e.g., transfer between bed and wheelchair), then sitting balance, verticalization, standing balance, and the walking. In this rehabilitation center, the aim of the therapy is too prevent falls in brain-damaged patients (SOFMER, 2010). The mechanical horse used in this study provided two-dimensional movements, anterior/posterior (i.e., forward/backward) and upward/downward. The amplitude of the anterior/posterior motion was 0.3 cm and the amplitude of the upward/downward motion was 11 cm, with the length of the mechanical horse being 174 cm. Moreover, the oscillation frequency was adjustable and ranged from 12.1 osc.min^−1^ (20% of the maximal oscillatory speed) to 150 osc.min^−1^ (100% of the maximal oscillatory speed) (Table 2). These frequencies can be similar to a real horse's gaits. Indeed, according to Galloux et al. (1994) and the Table 2, the trot (about 1.3 Hz) is similar to 60% frequency, the gallop (about 1.8 Hz) is similar to 90% frequency and therefore the frequencies from 20 to 50% are similar to walk of real horse.

**Table 2:** Horse's oscillation frequencies.

**% Horse oscillation speed**	**Oscillation frequencies (osc.min^**1**^)**	**Oscillation frequencies (Hz)**	**Amplitude**
20	12.1	0.2	Anterior/Posterior +/–0.3 cm Upward/Downward +/– 5.5 cm
30	26.5	0.44	
40	41.4	0.69	
50	57.7	0.96	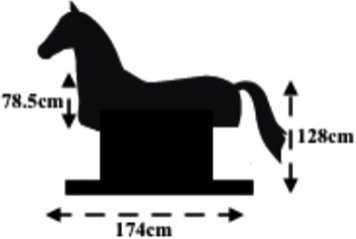
60	74.1	1.24	
70	88.2	1.47	
80	103.4	1.72	
90	125	2.08	
100	150	2.5	

**Figure 1:**
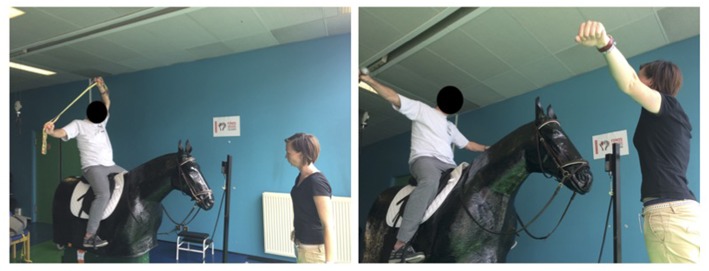
Example of exercises during training sessions.

Several works (Hosaka et al., 2010; Benoit, 2011; Han et al., 2012; Kang et al., 2012; Song et al., 2013; Sintim, 2014; Cho and Cho, 2015; Kang, 2015) have shown positive effects of using a riding horse simulator in the rehabilitation but these tools were different from that used here (i.e., previous tool had more than one degree of freedom). During exercise on the mechanical horse, the horse-riding instructor required participants to maintain sit up straight posture. The patients performed several exercises of balance and trunk mobilization that consisted of movements on the mechanical horse (e.g., arm movements: to the front, to the sides and upward; trunk movements: leaning forward, to the sides and backward; and movements with a ball). The arm, flexion-extension of the trunk, and leg exercises helped the patients to build muscle strength. The oscillation frequency of this tool was controlled by the instructor, according to the abilities and comfort level of each participant (Figure 1).

The study protocol was composed of a pre-test (in the first session), 24 training sessions (twice a week for 12 weeks; on the mechanical horse for the horse group, and physiotherapy sessions for control group), and a post-test (the last session). All participants of the horse and control groups performed the pre- and post-tests, which were composed of: (i) sitting on the horse during a rest period (3 min without horse movements) and then (ii) staying on the horse for several increments of oscillation frequencies (30, 40, 50%, etc.; maintained at least 1 min and 30 s), according to the abilities of each patient. According to dynamic approach, the incrementation of these oscillation frequencies is necessary in order to have the greatest possible range of postural coordination and observe the potential transitions between postural coordination in patients (Bardy et al., 1999, 2002; Bardy, 2004; Baillet et al., 2017b).

### Data Analysis

All data were collected in the medical rehabilitation hospital (CRMPR Les Herbiers, France).

#### Primary Outcome Measures

During the pre- and post-tests, the postural coordination of each patient was recorded with the OptiTrack system (Natural Point, Corvallis, OR, USA) (Thewlis et al., 2013). Ten cameras (100 Hz) compose this optokinetic system, which is based on the recognition of markers by infrared reflection. All participants were equipped with four reflective markers (i.e., head, second and seventh cervical vertebrae: C2, C7, and fifth sacral vertebra: S1). A last reflective marker was positioned on the mechanical horse (i.e., behind the saddle). Two angles were determined by the point coordinates of these markers for analysis of the discrete relative phases (DRP) between the participants and the mechanical horse. The angles were segmental and were calculated between two point coordinates and the vertical axis: the *head angle* calculated by the point coordinates of the head and C2, expressed as a function of the vertical axis, and the *trunk angle* calculated by the point coordinates of C7 and S1, expressed as a function of vertical axis. Similarly, the marker positioned on the horse and the vertical axis characterized the *horse angle*. We recorded the angular data series from the oscillatory movements of the mechanical horse, following sinusoidal oscillators: *head, trunk*, and *horse*. The angular positions of each body oscillator were compared to those of the horse's oscillator to compute the DRP between the head and horse and between the trunk and horse. The DRP is the difference in time between two similar occurrences from two oscillators, reported on the basis of the period of one cycle as a reference (Zanone and Kelso, 1992). In the present study, this reference was the horse's oscillator. Two relative phases were computed: ϕ_Head−Horse_, ϕ_Trunk−Horse_.

The ϕ_*rel*_ for the 18 patients were analyzed using Matlab version 8.3 (Matlab, 2014). At each oscillation frequency, a period of 30 oscillations was determined. This period corresponded to 15 values preceding and succeeding the central value of the trial. The ϕ_*rel*_ were computed in degrees: *in-phase* coordination between the patient and the horse was characterized by 0° ± 30° and 360° ± 30°, *antiphase* coordination was characterized by 180° ± 30°, and the other values represented *out-of-phase*.

#### Secondary Outcome Measures

In order to evaluate the change in postural coordination after the rehabilitation period, we subtracted the pre-test values from the post-test values (i.e., change = RP_post−test_-RP_pre−test_). Moreover, the standard deviation computed from 30 relative phases performed in each patient and in each condition, was used to estimate the variability.

### Statistical Analysis

The ϕ_trunk/horse_ and ϕ_head/horse_ were circular data (i.e., 0°-360°), where 0° and 360° represented the same orientation and the same polar angle. We therefore needed to use circular statistics (Batschelet, 1978), but circular statistics do not allow the computation of interactions between factors. In order to perform linear statistics, the range of ϕ_*rel*_ values was thus decreased to 0°-180°. Indeed, when the range of distribution of values is <180°, the difference between circular and linear methods is negligible (Pellegrini et al., 2004; Faugloire et al., 2006, 2009). The range was also reduced in Hodges and Franks (2002): all ϕ_*rel*_ values higher than 180° were subtracted from 360°.

Then, the statistical analysis was conducted with a three-way ANOVA: 2[Group_(Control/Horse)_] × 2[Rehabilitation_(Pre−test/Post−test)_] × 4[Frequency_(30%/40%/__50%/60%)_], with repeated measures (on frequency and rehabilitation) on the means of ϕ_*head*−*horse*_ and ϕ_*trunk*−*horse*_, and on their standard deviation. Moreover, statistical analysis of the changes in the postural coordination (and its variability) was conducted with a two-way repeated measures ANOVA: 2[Group_(Control/Horse)_] × 4[Frequency_(30%/40%/50%/60%)_].

For all analyses, the statistical threshold was established at *p* = 0.05. When the Mauchly test for sphericity was significant, the Greenhouse-Geisser correction was applied. To test for significant differences between the means (and standard deviation) of the factors (groups, rehabilitation and frequencies), the Bonferroni method was used for all *post-hoc* comparisons.

## Results

### Postural Coordination: Trunk/Horse

**Figure 2:**
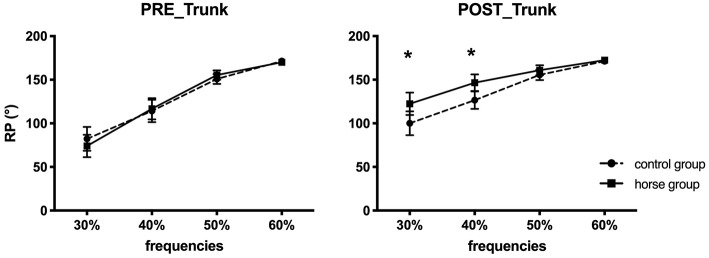
Depiction of trunk/horse relative phase (mean ± standard-error) in the pre- and post-tests, for the control (dashed line) and horse (continuous line) groups. ^*^Characterizes significant differences between groups.

The statistical analysis of ϕ_trunk/horse_ indicated (Table 3):

- A significant effect of rehabilitation: *F*_(1, 15)_ = 16.6 (*p* < 0.05). This major effect showed a significant modification in trunk/horse coordination between pre- and post-tests for all patients, although no real pattern change was observed. The mean ϕ_*rel*_ measured in pre-test was 129.4° ± 5.1°, characterizing *out-of-phase* coordination, while in post-test the coordination was modified and reached 144.5° ± 4.9°, again characterizing *out-of-phase* coordination but moving toward an *antiphase* coordination between patient trunks and the horse.
- A significant effect of the horse oscillation frequency: *F*_(1.8, 27.7)_ = 59.9 (*p* < 0.05). The Bonferroni test on the oscillation frequency showed that patient trunk/horse coordination was different for each frequency (30 ≠ 40 ≠ 50 ≠ 60%). The RP values revealed a change in the coordination pattern, from *out-of-phase* to *antiphase*, when the oscillation frequencies increased.
- However, no effect of group (*p* > 0.05) was observed for the ϕ_trunk/horse_ variable.
- An interaction effect of rehabilitation × frequency: *F*_(2.2, 32.4)_ = 13.7 (*p* < 0.05). The *post-hoc* tests showed that trunk/horse coordination presented a significant difference between pre- and post-test only when oscillation frequencies were low: at 30 and 40%. In contrast, the more the frequencies increased, the more similar the means of trunk/horse coordination became between pre- and post-test, moving from *out-of-phase* to *antiphase* coordination with the horse.
- An interaction effect of rehabilitation × frequency × group: *F*_(3, 13)_ = 3.1 (*p* < 0.05). The Bonferroni test showed that trunk/horse coordination was significantly different between pre- and post-test when oscillation frequencies were low (30 and 40%), only for patients of the horse group. These results indicate a change in the pattern of trunk/horse coordination of these patients after 12 weeks of rehabilitation (Figure 2), more specifically for low frequencies, which significantly differentiated the rehabilitation with and without mechanical horse.

**Table 3:** Relative phase (mean ± SD) and statistical results of the trunk/horse coordination for the two groups, at each oscillation frequency and in pre- and post-tests.

**Trunk/horse coordination**													
	**30%**			**40%**			**50%**			**60%**			**Means**
	**Control group**	**Horse group**	**Means**	**Control group**	**Horse group**	**Means**	**Control group**	**Horse group**	**Means**	**Control group**	**Horse group**	**Means**	
Pre-test	82.32°± 13.7°	74.09°± 12.9°/^*^	78.2°± 9.4°/^*^	114.2°± 12.9°	116.7°± 12.2°/^*^	115.4°± 8.9°	150.9°± 5.7°	155.5°± 5.4°	153.2°± 3.9°	171.5°± 1.9°	170.1°± 1.8°	170.8°± 1.3°	129.4°± 5.1°/^*^
Post-test	100°± 13.7°	122.4°± 12.9°/^*^	111.2°± 9.4°/^*^	126.5°± 32°	146.6°± 25.1°/^*^	136.6°± 7°	155.6°± 6.1°	161°± 5.2°	158.3°± 4.2°	171.2°± 1.4°	172.5°± 1.3°	171.8°± 0.9°	144.5°± 4.9°/^*^
Means	94.7°± 8.7°^**^			126.0°± 7.5°^**^			155.8°± 3.5°^**^			171.2°± 0.9°^**^			

** *Significant differences between all frequencies*.

/* *Significant differences between pre- and post-tests*.

### Postural Coordination: Head/Horse

**Figure 3:**
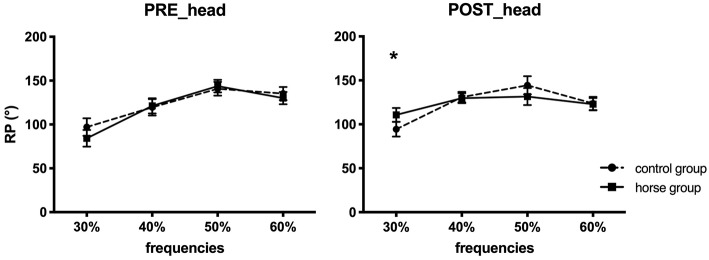
Depiction of relative phase (mean ± standard error) of head/horse coordination in the pre- and post-tests, for the control group (dashed line) and the horse group (continuous line). ^*^Represents significant differences.

The ANOVA performed on ϕ_head/horse_ revealed (Table 4):

- A significant effect of oscillation frequency: *F*_(1.7, 25.7)_ = 0.2 (*p* < 0.05). The Bonferroni test presented significant differences according to oscillation frequency. Indeed, at 30%, the coordination values were different from values measured at 40, 50, and 60%. In addition, RP values at 50% frequency were significantly different from those at 60%. Last, changes were observed in head coordination as a function of frequency, always in *out-of-phase* with the horse.
- However, no significant effect was observed between the two rehabilitation groups (*p* < 0.05) for head/horse coordination.
- Two interaction effects: rehabilitation x frequency: *F*_(3, 13)_ = 5.5 (*p* < 0.05) (Table 4), and rehabilitation × frequency × group: *F*_(3, 13)_ = 3.6 (*p* < 0.05) (Table 4; Figure 3). When *post-hoc* tests were performed on the interaction effect of rehabilitation × frequency, significant differences were observed between the head/horse coordination measured in pre- and post-test, only for the lower and higher frequencies (30 and 60%). For these frequencies, the head coordination of the patients was modified (always in *out-of-phase* as a function of the horse) after rehabilitation. Moreover, Bonferroni tests were performed on the interaction effect of rehabilitation × frequency × group and showed only one significant difference between pre-test and post-test for the horse group, at the lowest frequency (30%) (Table 4). Patients with rehabilitation on the mechanical horse were able to modify their head coordination pattern when the horse oscillated at 30% (Figure 3). No difference was shown for the control group patients.

**Table 4:** Relative phase (mean ± SD) and statistical results of the head/horse coordination for the two groups, at each oscillation frequency and in pre- and post-tests.

**Head/horse coordination**												
	**30%**			**40%**			**50%**			**60%**		
	**Control group**	**Horse group**	**Means**	**Control group**	**Horse group**	**Means**	**Control group**	**Horse group**	**Means**	**Control group**	**Horse group**	**Means**
Pre-test	97°± 10.1°	84.19°± 9.6° /^*^	90.6°± 7° /^*^	119.6°± 9.3°	121.2°± 8.8°	120.4°± 6.4°	140.8°± 7.8°	143.8°± 7.3°	142.2°± 5.4°	135.2°± 7.6°	130.1°± 7.1°	132.6°± 5.2°/^*^
Post-test	94.4°± 8.4°	110.7°± 7.9° /^*^	102.6°± 5.7° /^*^	130.8°± 6.1°	129.7°± 5.7°	130.3°± 4.2°	144.5°± 10.3°	131.6°± 9.7°	138°± 7.1°	123.8°± 7.5°	122.9°± 7°	123.3°± 5.1°/^*^
Means	*96.6°*± 6.1°^**^			125.3°± 3.9°			140.1°± 4.9°^*^			128°± 4.8°^*^		

** *Significant differences: 30% ≠ 40, 50, and 60%*;

* *Significant differences: 50 ≠ 60%*.

/* *Significant differences between pre- and post-tests*.

### Change in Postural Coordination (Post-Test–Pre-Test): Trunk/Horse, Head/Horse

#### Change in ϕ_Trunk/Horse_

**Figure 4:**
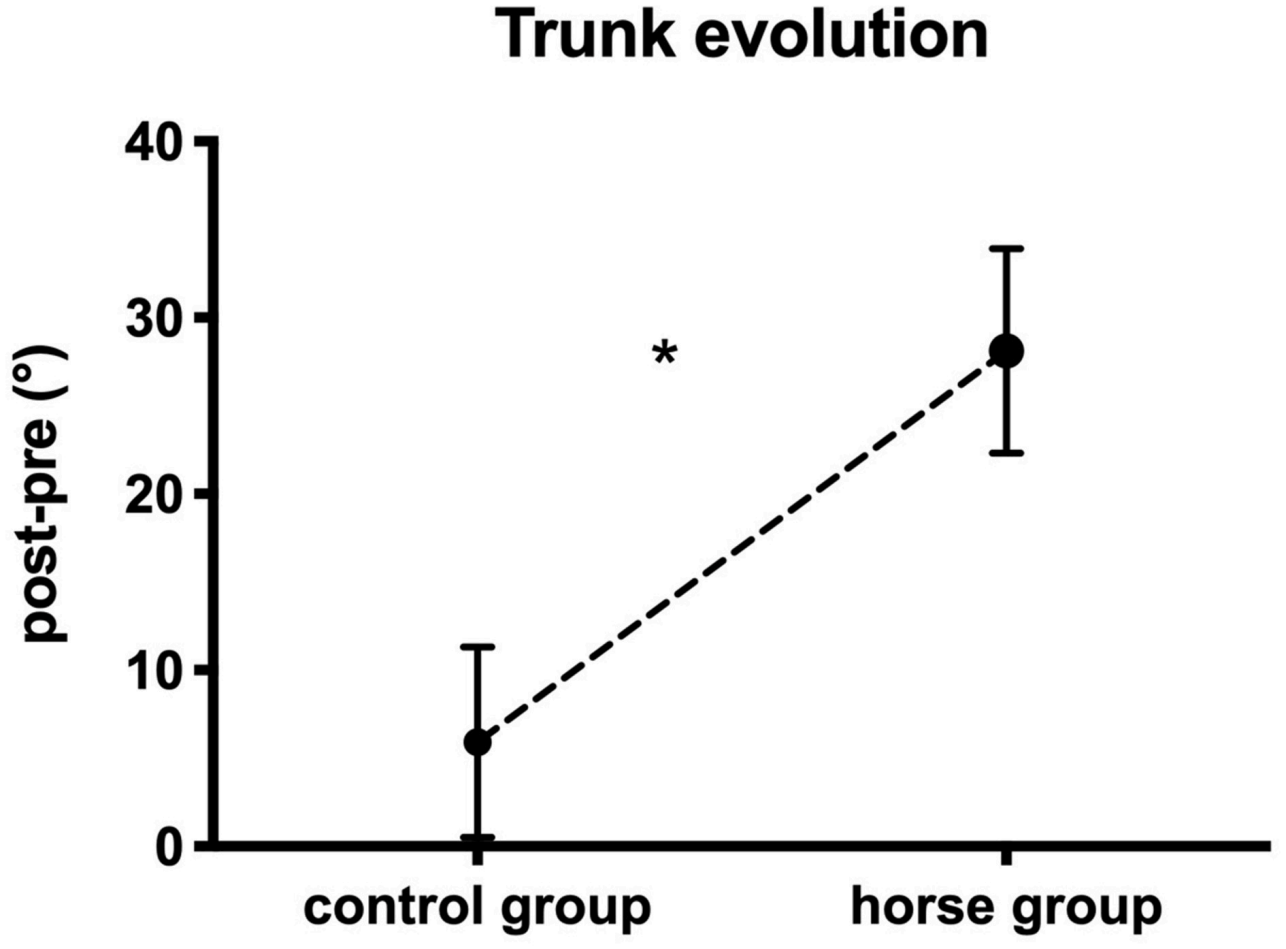
Mean change (post–pre; ±SE) in trunk/horse coordination for control and horse group. ^*^Represents significant difference between two groups.

The statistical analysis of the change in trunk/horse coordination (post–pre) indicated (Figure 4):

- A significant effect of oscillation frequency: *F*_(1.8, 19.6)_ = 11.48 (*p* < 0.05). The *post-hoc* tests to characterize the effect of oscillation frequency showed a change in trunk/horse coordination that differed as a function of the oscillation frequencies. Indeed, the change at 30% (37.1° ± 8.7°) differed from that at 50% (7.8° ± 3.9°) and 60% (2.1° ± 1.7°). Likewise, the change at 40% (21° ± 5.7°) differed from that at 50 and 60%. As the oscillation frequency increased, the postural change became smaller. For example, at 60% frequency, the postural coordination in pre-test was similar to the postural coordination in post-test.
- A significant effect of group: *F*_(1, 11)_ = 7.88 (*p* < 0.05). The Bonferroni test showed that the change in trunk/horse coordination was significantly greater, toward *antiphase*, for the horse group patients than the control group patients. Indeed, a change of 5.9° ± 5.4° was observed for control group vs. 28.1° ± 5.8° for horse group.

Despite the non-significant interaction effect between these two variables, all the results seem to indicate a greater postural change of the trunk toward *antiphase*, at low frequencies contrary to high frequencies, principally for the horse group (30%: 58.8° ± 26.9°; 40%: 36.3° ± 19.2°; 50%: 13.4° ± 15.6°; 60%: 3.7° ± 7.3°).

#### Change in ϕ_Head/Horse_

Statistical analysis of the progression of head/horse coordination indicated:

- An effect of oscillation frequency *F*_(3, 13)_ = 4.89 (*p* < 0.05). The *post-hoc* Bonferroni tests showed that the change in head/horse coordination differed as a function of oscillation frequency. A striking difference was observed between the 30 and 60% frequencies for all patients, indicating that it was positive at 30%, equal to 12° ± 4.1°, and negative at 60%, equal to −7° ± 2.6°. Indeed, the RP values of head/horse coordination increased in post-test at low frequencies and decreased (toward *in-phase*) with higher frequencies.
- An interaction effect of frequency x group: *F*_(3, 13)_ = 4.01 (*p* < 0.05). A significant difference was observed for the change in head/horse coordination, distinguishing the two rehabilitation groups at 30% frequency (horse group, at 30%: 26.5° ± 20.8°; at 40%: 8.6° ± 32.1°; at 50%: −11.5° ± 41.2°; at 60%: −1.9°± 10.4°).

### Variability of Postural Coordination

#### Standard Deviation of ϕ_Trunk/Horse_

Results of the ANOVA of ϕ_trunk/horse_ showed a significant effect of oscillation frequency: *F*_(2, 29.7)_ = 13.9 (*p* < 0.05). The *post-hoc* Bonferroni tests showed that the variability of trunk/horse coordination differed with the oscillation frequency. A considerable decrease in postural variability was observed when the frequency was increased. The variability measured at 30% (23.7° ± 12.3°) was significantly different from that at 50% (10.6° ± 7.2°) and 60% (5.5° ± 2.5°), and the one at 40% (18.2° ± 11°) was different from that at 60%. Last, the variability at 50% was different from the variability at 30 and 60%.

**Figure 5:**
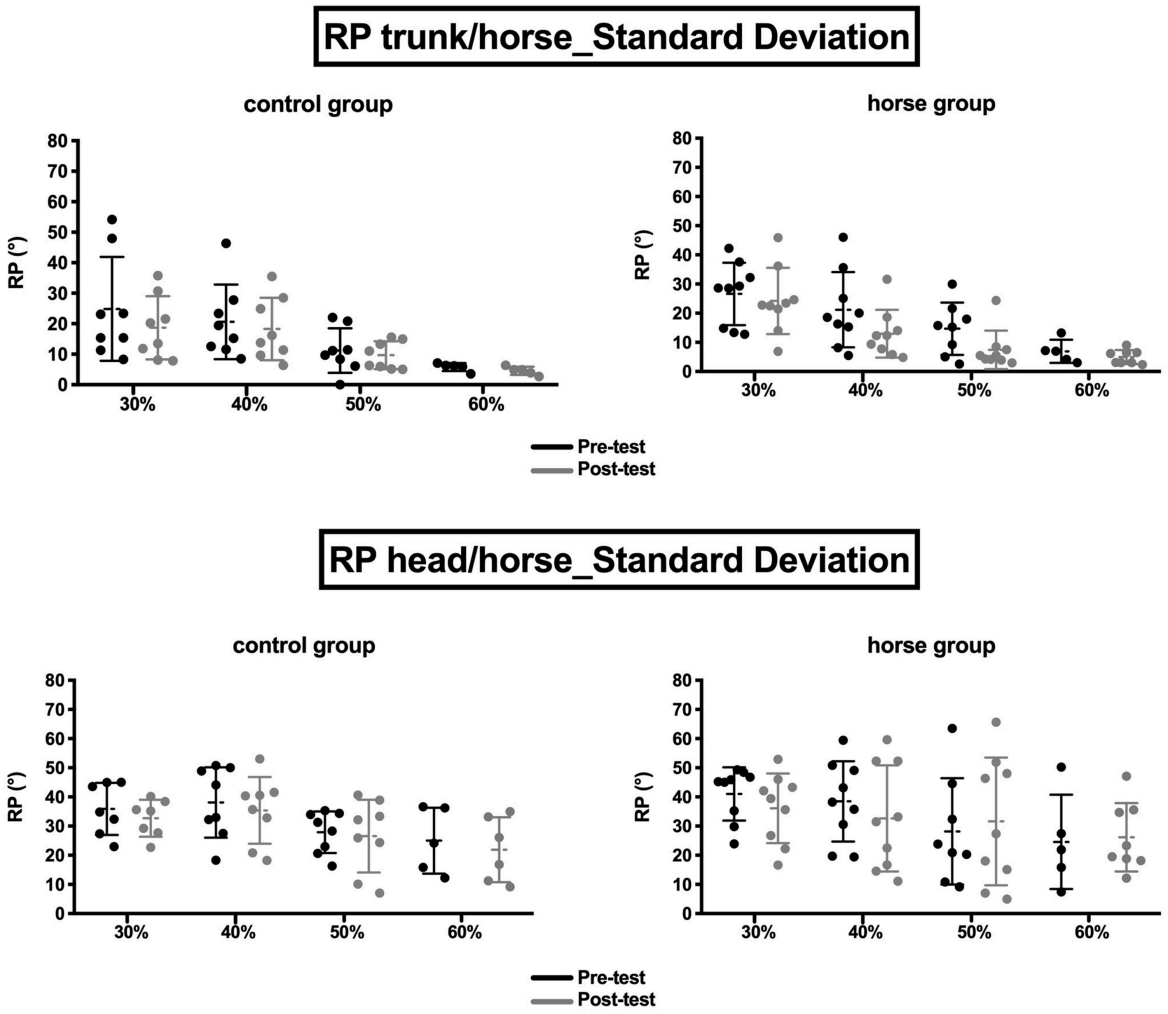
Relative phase variability (one point per patient; mean ± standard deviation) of trunk/horse **(Top)** and head/horse **(Bottom)** coordination, for the control **(Left)** and horse **(Right)** groups during pre-tests (in black) and post-tests (in gray), at each oscillation frequency.

The variability of the trunk/horse coordination values decreased between pre-test and post-test for the brain-damaged patients and for each oscillation frequency (e.g., for the horse group, at 50%: 14.7° ± 9.0° in pre-test and 7.4° ± 6.6° in post-test; for the control group, 11.2° ± 7.3° in pre-test and 9.7° ± 4.5° in post-test) without significantly distinguishing the two rehabilitation groups (control and horse) (Figure 5).

However, neither a significant group effect (*p* > 0.05) nor a significant interaction effect (*p* > 0.05) was observed.

#### Standard Deviation of ϕ_Head/Horse_

As for trunk/horse coordination, the statistical analysis of the RP standard deviation of head/horse coordination showed a single significant effect of oscillation frequency: *F*_(3, 14)_ = 5 (*p* < 0.05). The *post-hoc* tests showed a decrease in the variability of this coordination as a function of oscillation frequency. The variability measured at 30% (23.7° ± 12.3°) was different from that at 60% (5.5° ± 2.5°), and the one at 40% (18.2° ± 11.0°) was also different from the variability at 60%.

Nevertheless, the change in variability values seemed to be slightly more pronounced for the control group concerning this coordination (Figure 5).

However, neither a group effect (*p* > 0.05) nor an interaction effect was observed (*p* > 0.05).

#### Change in Postural Variability (Post–Pre)

##### Standard deviation of trunk/horse RP change

The ANOVA of the change in trunk/horse RP standard deviation did not show a significant effect of rehabilitation group (*p* > 0.05) or frequency or an interaction between these variables (*p* > 0.05).

##### Standard deviation of head/horse RP change

Similarly, statistical analysis of the change in head/horse RP standard deviation revealed no significant effect of frequency, group or interaction between the two groups (*p* > 0.05).

## Discussion

Using of this tool is new in rehabilitation centers and few studies have demonstrated its effectiveness (Baillet et al., 2017a,b, 2018). Therefore, the aim of this pilot study was to evaluate the impact of a new training protocol using a mechanical horse on the postural coordination of brain-damaged patients.

### Changes in Trunk/Horse and Head/Horse Coordination

A change in trunk/horse coordination was observed for all patients after 12 weeks of training, with or without the mechanical horse. However, although the difference between pre-test and post-test coordination was significant for all patients, a real change in trunk/horse coordination mode was not observed as it remained *out-of-phase* despite approaching *antiphase* in the post-test. Previous studies have shown that *antiphase* coordination between trunk and horse is the spontaneous coordination adopted by riders on a horse (Lagarde et al., 2005; Ancelet, 2006) and non-riders (healthy individuals) on a mechanical horse (Baillet et al., 2017b). This antiphase coordination can be characterized as a strong attractor (Zanone and Kelso, 1992; Bardy et al., 1999, 2002; Lagarde et al., 2005). The postural change we observed indicated only a change in coordination toward the appropriate pattern (*antiphase*, characterizing pelvis/trunk dissociation on the horse), but it remained *out-of-phase*. However, these results concern the means for all 18 patients, without differentiating the two rehabilitation groups, and this may explain why we did not observe a shift to *antiphase*. Moreover, the change in head/horse coordination after 12 weeks of training was not significant in these patients. In pre-test and post-test, *out-of-phase* coordination between head/horse was observed with no change in coordination pattern (121.5° ± 3.7° in pre-test against 123.5° ± 4.6° in post-test).

### Coordination Changes According to Oscillation Frequency

In both groups, postural coordination depended on the oscillation frequency of the mechanical horse. Trunk/horse coordination was different at each oscillation frequency, evolving toward the *antiphase* pattern when the frequency increased (171.2° ± 0.9° at 60%). Likewise, head/horse coordination presented significant differences as a function of oscillation frequency, but these changes were less pronounced than for trunk/horse coordination.

Despite the changes in coordination pattern, patient heads were always *out-of-phase* in relation to the horse, whatever the oscillation frequency. The environmental constraints imposed by the horse's rhythmic movements seemed to act differently on the patients' postural segments. The trunk was closest to the horse, being directly in contact with the saddle. It is possible that the movements imposed by this tool had a direct impact on the existing coordination between the patients' trunk and the horse itself (Lagarde et al., 2005; Ancelet, 2006), which also might have made it easier for the patients to adapt to the horse's movements (Newell, 1986). The head, however, was not directly in contact with the horse but acted as a prolongation of the trunk, and the horse's movements seemed to be reflected in an offset manner for the head/horse coordination. This may have made adaptation more difficult, especially in our brain-damaged patients. Several experiments have shown that during walking the head is an inertial platform and that its stabilization in space serves as a basis for the descending organization of postural control (Grossman et al., 1988; Pozzo et al., 1990; Winter, 1995, 2009; Nicholas et al., 1998; Mulavara et al., 2002; Vasseur, 2015). According to these authors, head/trunk coordination is also an articulated set. Nevertheless, postural analysis in children (Vasseur, 2015), the elderly (Brand, 1992) and patients (Winter, 1991) has revealed that this notion of an articulated set is inaccurate, and these authors instead presented the notion of an “in-block coordination” between head/trunk segments. This in-block coordination may correspond to a freezing of the degrees of freedom as stated by Bernstein (1967). This would explain our results, given that the coordination of our patients' heads was *out-of-phase* with the horse (yet approaching *antiphase* at higher frequencies), preventing the latter from achieving adequate coordination on a horse: *in-phase* (Lagarde et al., 2005; Ancelet, 2006; Baillet et al., 2017b; Olivier et al., 2017). The second explanation is the head's essential role in controlling balance because it contains the necessary visual and vestibular systems (Winter, 1991; Mulavara et al., 2002; Nadeau et al., 2003). The vestibular system in animals has been shown to be specifically concerned with equilibrium control during locomotion tasks inducing high levels of imbalance (Marchand and Amblard, 1990; Nadeau et al., 2003). In this study, the vestibular system of the patients seemed to be impacted by the horse's most and least rapid movements, which endangered the balance of their heads. Moreover, the visual information needed by the patients during the pre- and post-tests was restricted because the horse was located in a rehabilitation room with windows that had been caulked. It may have been more difficult for the patients to adapt and maintain a head position in balance with the horse under these conditions. The role of the environment is determinant and additional visual information might have had a different impact on head/horse coordination (e.g., a horse outdoors), explaining why the patients' head/horse pattern remained *out-of-phase* whatever the oscillation frequency, whereas the trunk/horse pattern joined the main attractor of this coordination in *antiphase* (Zanone and Kelso, 1992).

### Coordination Changes According to Group and Oscillation Frequency

An interaction effect for trunk/horse coordination was observed only for the horse group: a change in postural pattern between the pre- and post-tests for the lower horse oscillation frequencies (30 and 40%). This result differentiated our two groups and showed the interest of the mechanical horse in a postural rehabilitation protocol, validating the recent studies carried out on this tool (Kubota et al., 2006; Han et al., 2012; Song et al., 2013; Sintim, 2014; Cho and Cho, 2015; Kang, 2015). Indeed, the coordination values measured during our two evaluations showed a change in trunk/horse coordination toward *antiphase* even when the frequency was low. For the control group, the average RP values indicated a non-significant change in this coordination in post-test (e.g., at 40%: 114.2° ± 42.4° in pre-test, 126.5° ± 32.0° in post-test). However, the functional aspect of these results for the control patients (who performed only the 2 evaluative sessions on this tool) can be questioned. The pre-test was the very first use of the mechanical horse for all patients. Although the control patients only had two sessions on the horse, even brief familiarization with it may have prompted a change in coordination. Indeed, apprehension and complete unfamiliarity in the first session may have made the patients tense and static at the different frequencies, whereas during the post-test (second evaluation) they were more attuned to its functioning (e.g., its frequencies). However, without real learning and training sessions on the tool, this did not enable them to reach adequate coordination (Nourrit et al., 2003). We observed similar results for head/horse coordination. Only one significant difference between the pre- and post-tests was observed, only for the horse group patients at the lowest frequency, 30%. In other words, mechanical horse training helped these patients to change their head coordination pattern when the horse oscillated at 30% (84.2° ± 29.5° in pre-test; 110.7° ± 18.8° in post-test), in contrast to the patients of the control group. The essential role of the training sessions on the mechanical horse was thus shown by the behavioral changes in the brain-damaged patients. Indeed, the 24 sessions enabled them to adapt to the task constraints (Newell, 1986) on the horse through coordination changes.

### Postural Coordination Changes

The changes in the two postural coordination were measured in order to compare the two groups for these changes after 12 weeks of training. In the first step, the change in head coordination with the horse was significantly better for patients after 24 sessions at 30% frequency. In the second step, the change in trunk/horse coordination was measured and significantly differentiated the two groups because the coordination pattern was substantially changed in the post-test for the patients of the horse group. These results are consistent with Park et al. (2013)'s conclusion of better postural adjustment of the trunk of brain-damaged patients after rehabilitation on an equestrian simulator. The horse group patients clearly were able to adapt to the task constraints (Newell, 1986) after 24 sessions on the mechanical horse, as they were able to organize and recall the posture of a rider evolving on the horse (Lagarde et al., 2005; Terada et al., 2006; Byström et al., 2015). In other words, the brain-damaged patients learned through training on the horse, modifying their spontaneous postural coordination during sessions in order to achieve *antiphase* coordination between the trunk and horse. This rehabilitation technique gave them the ability to develop specific coordination methods to optimize their posture (Megrot and Bardy, 2005).

### Coordination Variability and Changes in Variability

However, the statistical analysis of the variability in postural coordination (measured by the coordination standard deviation and the change in this standard deviation after rehabilitation) did not permit to differentiate the two types of training. Whether for head/horse or trunk/horse coordination, a significant decrease in variability was observed for all patients based on the horse's oscillation frequency. As a result, we noted greater stability in these patients when the frequencies were higher (e.g., 60%), which may have been related to the patients' determinism (Riley and Turvey, 2002) or the attention cost when the horse's movements increased (Kahneman, 1973).

Last, the analysis of the change in variability (post–pre) showed no significant distinction between the two training methods. A detailed analysis of the values (and graphs) nevertheless revealed a trend suggesting a change toward more negative values for the control group and more positive values for the horse group. In other words, control group patients presented values of postural variability lower in post-test than in pre-test, indicating a decrease in variability [and thus increased stability (Newell et al., 1993)], whereas the horse group patients also presented a decrease in this variability post-test but the values remained higher than in pre-test. The brain-damaged patients who performed 24 sessions on the mechanical horse thus showed greater postural variability post-test than the control group, which enabled them to adapt more easily to the task constraints, and this was the case for every oscillation frequency. According to Stergiou and Decker (2011), the postural variability of these patients does not necessarily mean instability but rather flexibility. A theoretical model was developed by Harbourne and Stergiou (2009) to explain movement variability in motor learning and health. This model is based on the idea that mature motor skills and health states are associated with optimal variability of movement that reflects the adaptability of the underlying control system (Harbourne and Stergiou, 2003, 2009; Stergiou and Decker, 2011). These authors specifically explained that chaotic behavior can appear following fatigue (or simply be related to pathology) even in, for example, a subject expert in the activity. Thus, postural variability can be impacted (increase in variability) but without affecting the individual's performance and therefore postural control (Stergiou and Decker, 2011). Indeed, expert behavior is flexible, showing adaptation and reduced stability, without postural coordination being impacted. Therefore, after 24 sessions of training on the mechanical horse, these brain-damaged patients of the horse group probably did not present novice behavior. Instead, through learning during the sessions, they became able to adapt their posture without impacting performance, which would explain this greater variability. Indeed, the motor cortex of a patient after rehabilitation is not the same as that of a healthy individual, since plasticity ensures different motor solutions for performing the same task (here, maintaining posture on the horse). This “ability of elements that are structurally different to perform the same function or yield the same output” (Edelman and Gally, 2001, p. 13763) is called degeneracy by Edelman and Gally (2001), and it may be responsible of the emergence of a patient's mode of coordination that is less deterministic and more oriented toward exploration, thus showing greater behavioral variability.

Furthermore, the exercise performed in Park et al. (2013)'s study on the simulator improved the functional balance of patients post-stroke, which we did not observe in our study. However, the equestrian simulator is similar to the Persival simulator (Jouffroy, 1991; Richard and Léard, 1993), and thus it identically reproduces the paces of a real horse. In our study, the simulator was very different from Park et al. (2013)'s—a mechanical horse with a single movement in the anterior-posterior plane—and this might explain the postural differences in the horse group of our brain-damaged patients.

## Limits

This study had several limitations, particularly related to: (1) the sample size (a shortfall of 18 patients) and the sample heterogeneity (traumatic brain injuries and post-stroke); (2) the risk of bias concerning blinding procedure of open label study (all parties, patients, and clinicians, were aware of the training method the participant receive); (3) the sample size and power analysis have not calculated because this is a preliminary study; (4) only the horse group's participants were undergone horse training; (5) the missing of clinical measures (these results were not used in this present article in order to emphasize on the postural coordination of patients).

## Conclusion

This study showed the interest of using the mechanical horse in training to improve the postural coordination of brain-damaged patients. After 12 weeks of training (24 sessions), the postural coordination of the horse group patients was better than that of the control group, highlighting their ability to adapt to constraints and develop specific modes of postural coordination (trunk/horse *antiphase*) in order to optimize their posture. However, this is a preliminary study with several limitations and without clinical relevance. Currently, further studies better designed needed to demonstrate the efficacy of this tool in the rehabilitation of patients with neurological disorders.

## Ethics Statement

This study was carried out in accordance with the recommendations of the human research ethics committee of Lille University with written informed consent from all subjects. All subjects gave written informed consent in accordance with the Declaration of Helsinki. The protocol was approved by the human research ethics committee of Lille University (n° 2016-1-S39).

## Author Contributions

HB, DL, EV, NB, JK, and RT contributed conception and design of the study. HB organized the database and wrote sections of the manuscript. CD allowed the inclusion of patients. All authors contributed to manuscript revision, read and approved the submitted version.

### Conflict of Interest Statement

The authors declare that the research was conducted in the absence of any commercial or financial relationships that could be construed as a potential conflict of interest.
