# Supplementary material for: Effect of Mechanical Horse Practice as New Postural Training in Patients With Neurological Disorders: A Pilot Study
Source: Front Psychol. 2019 May 8;10:1035. doi: 10.3389/fpsyg.2019.01035 (PMC6517495; doi:10.3389/fpsyg.2019.01035)
Supplement: Supplementary file 1 [file Data_Sheet_1.PDF]

Horse group										
**Patients**	*N°1*	*N°3*	*N°4*	*N°8*	*N°9*	*N°10*	*N°15*	*N°16*	*N°17*	*N°18*
**Stroke location**	Cerebral anoxia	Left capsulo-lenticular hematoma	Extra dural left parietal hematoma, cerebral edema with mass effect in midline	Right middle cerebral artery occlusion M1	Deep right capsulo-lenticular hematoma	Hemorrhagic stroke of the left midbrain with centricular contamination	Sylvian ischemic stroke left	Cerebellar hematoma and subarachnoid heamorrhage on aneurysmal rupture of right PICA	Left occipital intraparenchymal hematoma on rupture of an arteriovenous malformation	Left laterobulbar infarction, left PICA and left cerebellar artery
**Stroke date**	05-25-2016	01-28-2016	10-10-2015	04-19-2016	09-27-2016	01-31-2017	04-02-2017	03-31-2017	09-05-17	07-19-2017
**Pharmacological treatment**	Lovenox<br>Brilique<br>Kardegic<br>Atenolol<br>Tahor<br>Domperidone<br>Spasfon<br>Duphalac<br>Paracetamol<br>Peptamen	Lovenox<br>Perindopril<br>Loxen<br>Augmentin<br>Ogastoro<br>Nutrison<br>Paracetamol	Paroxetine<br>Levetira<br>Etam<br>Paracetamol	Kardegic<br>Tahor<br>Lovenox<br>Fluoxetine<br>Eupantol<br>Lyrica<br>Imovane<br>Paracetamol	Amlor<br>Augmentin<br>Tahor<br>Lovenox<br>Eupantol<br>Lyrica<br>Eupressyl<br>Imovane	Ceftria<br>Xone<br>Lovenox<br>Paracetamol<br>Acupan<br>Novorapid	Unspecified	Nutrison<br>Tanganil<br>Alprazolam<br>Azinc<br>Fluoxetine<br>Pantoprazole<br>Doliprane<br>Tramadol<br>Imovane<br>Lovenox<br>Scopoderm	Tardyferon<br>Alvityl<br>Paracetamol<br>Nutrison proteine + nutrison<br>lovenox	Aspegic<br>Lovenox<br>Pantoprazole<br>Tahor<br>Zophren<br>Tanganil<br>Perfalgan

Control group								
**Patients**	*N°5*	*N°6*	*N°11*	*N°12*	*N°14*	*N°19*	*N°20*	*N°21*
**Stroke location**	Right sylvian infarction on right internal carotid occlusion	Right protuberential paramedian infarction, sylvian left and junctional left anterior	Cerebral infarction of middle cerebral artery and right anterior cerebral artery	Right sylvian ischemic stroke	Right carotid giant aneuvrysm	Left temporal intra-parenchymal hematoma, meningeal hemorrhage of cerebellum tent	Intraparenchymatous hematoma of the cerebral trunk	Left sylvian infarction
**Stroke date**	08-11-2015	08-14-2016	02-07-2017	11-29-2016	05-18-2016	11-03-2016	08-30-2017	08-10-2017
**Pharmacological treatment**	Kardégic<br>Perindopril<br>Tahor<br>Eupantol<br>Prozac<br>Xanax<br>Paracétamol<br>Laroxyl	Coumadin<br>Bisoprolol<br>Ramipril<br>Pantoprazole<br>Speciafoldine<br>Tahor<br>Nicobion<br>Nutrison	Nimotop<br>Lovenox<br>Kardegic<br>Speciafoldine<br>Acupan<br>Paracetamol	Acide acetylsalicylique<br>Atorvastatine<br>Baclofene<br>Fluoxetine<br>Zolpidem<br>Paracetamol		Baclofene<br>inexium<br>paracetamol<br>Fresubin<br>Lovenox	Nutrison<br>Temesta<br>Eupantol<br>Paracetamol<br>Deroxat<br>Lovenox	Kardegic<br>Amlodipine<br>Atorvastatine
